# Mesenchymal stem cells modified with angiopoietin-1 gene promote wound healing

**DOI:** 10.1186/scrt324

**Published:** 2013-09-16

**Authors:** Yunling Li, Lei Zheng, Xia Xu, Lili Song, Yin Li, Wei Li, Suhan Zhang, Feng Zhang, Haiyan Jin

**Affiliations:** 1 Department of Dermatology, Children’s Hospital, School of Medicine, University of Zhejiang, 57 Zhugan Lane, Yanan Road, Hangzhou, Zhejiang 310013, China; 2 Zhejiang Key Laboratory for Diagnosis and Therapy of Neonatal Diseases, Children’s Hospital, School of Medicine, University of Zhejiang, Hangzhou, Zhejiang 310013, China

## Abstract

**Introduction:**

Treatment of chronic skin wounds is difficult and largely ineffective. Little improvement has been shown in promoting the healing of these wounds in the past few decades. Innovative treatments to enhance chronic wound healing process are therefore needed.

**Methods:**

In this study, we examined the efficacy of angiopoietin-1 gene-modified bone marrow mesenchymal stem cells (Ang1-MSCs) on the promotion of cutaneous wound healing in rats. Excisional full-thickness wounds were treated with Ang1-MSCs, a recombinant adenovirus encoding angiopoietin-1 (Ad-Ang1), unmodified bone marrow mesenchymal stem cells (MSCs), or vehicle medium (sham).

**Results:**

The results showed that Ang1-MSCs significantly promoted wound healing with increased epidermal and dermal regeneration, and enhanced angiogenesis compared with MSCs, Ad-Ang1 or sham treatment. Moreover, Ang1-MSCs expressed CD31 in the wound, suggesting a direct contribution of Ang1-MSCs to angiogenesis.

**Conclusions:**

Here we show that Ang1-MSCs accelerate wound healing by promoting skin regeneration and angiogenesis, compared with MSCs or Ad-Ang1 alone.

## Introduction

The wound-healing process involves complex biological and molecular events including cell migration, proliferation, extracellular matrix deposition, angiogenesis, and tissue remodeling. Angiogenesis is an essential step in the process, and the formation of new blood vessels ensures the delivery of oxygen, nutritional support and growth factors. However, angiogenesis is often markedly impaired in chronic wounds, and poor angiogenesis delays wound healing. Strategies to stimulate angiogenesis have therefore been investigated as an approach to accelerating wound healing [1].

Mesenchymal stem cells (MSCs) can differentiate into a variety of mesodermal cell lineages [2-6]. Recent studies found that MSCs significantly enhanced wound closure and showed satisfactory effects in the treatment of chronic wounds in humans and animals [7-18]. Further studies demonstrated that MSCs promoted wound healing by improving angiogenesis [19,20]. Moreover, some studies reported that engrafted MSCs in the wound exerted a paracrine effect on angiogenesis by increasing the levels of angiogenic factors such as angiopoietin-1 (Ang1) and vascular endothelial growth factor [21].

Angiopoietins represent a major family of angiogenic factors, including Ang1, which acts via the phosphorylation of the Tie-2 receptor [22]. The Ang1/Tie2 interaction mediates maturation of neovessels into more complex and functional vasculature [23,24]. Recent studies revealed that the application of recombinant adenovirus encoding angiopoietin-1 (Ad-Ang1) or Ang1 protein accelerates wound closure, skin regeneration, and angiogenesis in animals [25,26].

These studies showed that the application of MSCs alone, Ad-Ang1 or Ang1 protein could accelerate wound healing. However, MSCs alone do not produce enough Ang1, which promotes angiogenesis, while Ad-Ang1 or Ang1 protein alone do not provide the cells and cellular factors needed to create the optimal wound-healing microenvironment. Ang1 gene-modified MSCs may therefore help to overcome these problems. Angiopoietin-1 gene-modified bone marrow mesenchymal stem cells (Ang1-MSCs) not only continuously release Ang1 *in situ*, but also provide cells and cellular factors to create a suitable environment to lead to faster wound-healing. We therefore compared the rate of wound healing promoted by Ang1-MSCs, MSCs, and Ad-Ang1 in an excisional-wound model. We implanted green fluorescent protein (GFP)-expressing Ang1-MSCs into excisional wounds and examined their effects on skin regeneration and angiogenesis compared with MSCs, Ad-Ang1 or sham treatment. Our results showed that compared with MSCs, Ad-Ang1 or sham, Ang1-MSCs accelerate wound healing by promoting skin regeneration and angiogenesis.

## Materials and methods

### Isolation and purification of mesenchymal stem cells

Bone marrow was collected from the femurs of 5-week-old to 7-week-old male Sprague–Dawley rats. Bone marrow mononuclear cells were isolated by Ficoll–Paque density-gradient centrifugation. Nucleated cells were plated in plastic tissue culture dishes and incubated in Dulbecco’s modified Eagle’s medium (DMEM; Gibco, Grand Island, NY, USA) supplemented with 10% fetal bovine serum (Gibco) and 1% penicillin–streptomycin solution (Sigma-Aldrich, St Louis, MO, USA) at 37°C with 5% CO_2_. Confluent cells were trypsinized and expanded.

### Phenotypic characterization of mesenchymal stem cells

Cell immunophenotypes were examined by flow cytometric analysis and immunofluorescence staining. For flow cytometry, the cells were incubated at 4°C for 30 minutes with fluorescein isothiocyanate-conjugated monoclonal antibodies against CD34, CD45, CD29, and CD90 or control isotype IgG (all obtained from BD Pharmingen, San Diego, CA, USA). The labeled cells were analyzed using a flow cytometer with Cell Quest software (Becton Dickinson, CA, USA). For immunofluorescence staining, subconfluent cells were cultured on chambered slides and were then fixed with 4% paraformaldehyde. The samples were incubated with primary antibodies at 4°C overnight. The primary antibodies were detected using Cy2-conjugated or Cy3-conjugated secondary antibodies. Cell nuclei were stained with 4′,6-diamidino-2-phenylindole (Sigma-Aldrich).

### Generation of Ad-GFP-Ang1

Infectious recombinant adenovirus particles were collected after homologous recombination of a shuttle vector with the pAdEasy system (Qbiogene, Carlsbad, CA, USA) in BJ5183 cells and packaging in embryonal kidney 293 cells. Briefly, Ang1 cDNA was subcloned into the shuttle plasmid pAdTrack-CMV. The resultant plasmid was linearized by digestion with the restriction endonuclease *Pme*I and then transformed into *Escherichia coli* BJ5183 that had been transformed with the adenoviral backbone plasmid pAdEasy-1. Recombinant plasmids were screened with kanamycin and confirmed by restriction endonuclease analysis (*Pac*I). The recombinant adenovirus vector was packaged and amplified in 293 cells.

### Infection of mesenchymal stem cells

The MSCs were grown to 80% confluence and were then cultured in fresh nonserum media containing the recombinant adenovirus. After incubation for 2 hours, the medium was then removed and the cells were washed with DMEM and recultured in normal medium. To evaluate the transduction efficiency of Ad-GFP-Ang1 on MSCs, GFP was detected by fluorescence microscopy.

### Analysis of exogenous gene expression

Exogenous Ang1 gene expression in MSCs was detected using RT-PCR and western blot analysis. For RT-PCR, total RNA was extracted from cultured cells using TRIzol reagent (Invitrogen, Carlsbad, CA, USA) following the manufacturer’s instructions. Purified RNA was then used for one-step RT-PCR (Qiagen Corporation, Valencia, CA, USA). The Ang1 primers used were: 5′-AAATATGAAGTCGGAGATGG-3′ and 5′-CAAAGGCTGACAAGGTTATG-3′). Relative quantification of target-gene expression was performed using the housekeeping gene β-actin as an internal control.

For western blotting, cell proteins were extracted in a lysis buffer containing 1% Triton X-100 and proteinase inhibitors (Sigma-Aldrich). The protein concentrations were determined using the Bradford dye-binding assay with bovine serum albumin as the standard. Equal amounts of the protein and prestained molecular weight markers were separated on SDS-PAGE and transferred onto polyvinylidene difluoride membranes. The membranes were then incubated with a mAb against Ang1 (Abcam, Cambridge, MA, USA), followed by incubation with horseradish peroxidase-conjugated secondary antibody. The protein bands were detected by chemiluminescence using an ECL kit (Perfect Biotech, Shanghai, China).

### Animals and treatment

Male rats (8 to 10 weeks old) with an average weight of 250 to 300 g were used in this study. Animal care and experimental procedures were approved by the Animal Care Committees of Zhejiang University. Hair was removed from the dorsal surface and the animals were anesthetized. Two 2 cm diameter full-thickness excisional skin wounds were then created on each side of the midline. The rats were then divided randomly into the following groups: sham (treatment with an equal volume of vehicle medium, *n* = 10), MSCs (treatment with 1 × 10^6^ MSCs, 0.7 × 10^6^ in 80 μl DMEM injected intradermally at the margin of the excisional wound at four injection sites and 0.3 × 10^6^ in 20 μl DMEM applied onto the wound bed, *n* = 10), Ad-Ang1 (treatment with 1 × 10^9^ plaque-forming units of recombinant adenovirus expressing Ang1, *n* = 10), and Ang1-MSCs (treatment with 1 × 10^6^ GFP^+^-Ang1-MSCs, 0.7 × 10^6^ in 80 μl DMEM injected intradermally at the margin of the excisional wound at four injection sites and 0.3 × 10^6^ in 20 μl DMEM applied onto the wound bed, *n* = 10).

### Evaluation of wound closure

The percentage of wound closure was calculated as follows:

$$\begin{array}{c} (\text{Area of original wound - area of actual wound}/\\ \;\text{area of original wound})\times 100 \end{array}$$

The wound area was measured by tracing the wound margin followed by calculation using an image-analysis program. The digital analysis was carried out by investigators who were blinded to group assignment.

### Histochemistry and immunohistochemistry

The rats were sacrificed at 3, 7, 14, and 21 days after the different treatments, at which time points skin samples, including the wound and the surrounding skin, were harvested using punch biopsy. Freshly harvested samples were fixed and embedded for histochemical analysis. The sections were stained with hematoxylin and eosin.

For immunohistochemical labeling, sections were incubated overnight at 4°C with primary antibody, then incubated with biotinylated secondary antibody, followed by incubation with streptavidin–horseradish peroxidase. Antibody binding sites were visualized by incubating in a diaminobenzidene solution, and counterstained with hematoxylin.

For Masson’s trichrome staining, the tissue sections were deparaffinized and rehydrated, and were then stained according to the Masson’s Trichrome Staining Kit (Yike Biotech, Guangzhou, China) instructions. Briefly, sections were placed in Bouin’s fixative after being deparaffinized and rehydrated through xylene and ethanol, and stained in Weigert’s iron hematoxylin working solution, followed by staining in Biebrich scarlet acid fuchsin solution. The sections were then differentiated in phosphotungstic acid solution and transferred directly (without rinsing) into light green solution. After rinsing, the sections were differentiated in 1% acetic acid solution, followed by dehydration in 95% ethyl alcohol and absolute ethyl alcohol. This method allows for the collagen to be stained green.

### Immunostaining and confocal microscopy

Double-staining was performed to determine whether MSCs differentiated into endothelial cells and whether the transplanted cells expressed Ang1 in the wound. The tissue sections were preincubated with sodium borohydride to reduce autofluorescence and were then incubated with a mAb against CD31 (Novus Biologicals Inc., Littleton, CO, USA) or Ang1, which was followed by detection with a Cy3-conjugated secondary antibody. The MSCs were tagged with GFP and therefore did not need to be labeled for easy visualization. The nuclei were stained with Hoechst. Confocal images were obtained using a Zeiss LSM 510 confocal microscope (Carl Zeiss, Jena, Germany).

### Statistical analysis

Data were expressed as the mean ± standard deviation and were analyzed by one-way analysis of variance followed by a Bonferroni’s *post-hoc* test for multiple comparisons. *P* <0.05 was considered statistically significant.

## Results

### Characteristics of primary antibody and Ang1-MSCs

**Figure 1:**
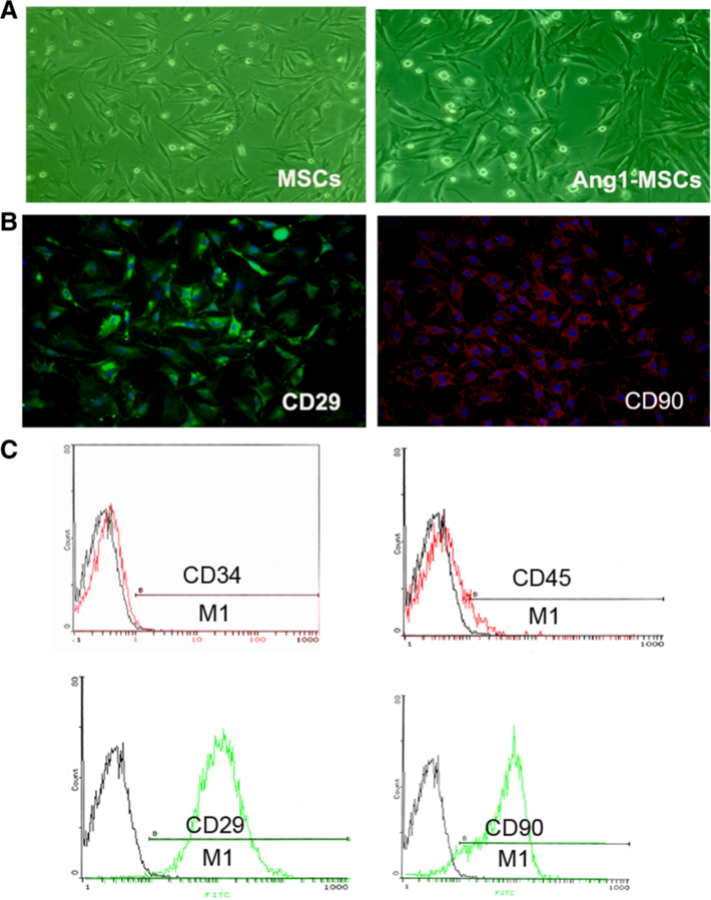
**Characterization of mesenchymal stem cells. (A)** Morphologies of mesenchymal stem cells (MSCs) and angiopoietin-1 gene-modified bone marrow mesenchymal stem cells (Ang1-MSCs) in culture. **(B)** Immunophenotypes of MSCs in culture. **(C)** MSCs were analyzed using flow cytometry.

Both flattened and spindle-shaped cells were present in culture. Ang1 gene-modified MSCs showed similar flattened and spindle shapes in culture (Figure 1A). Immunophenotypic analysis showed that the MSCs used in the experiment were negative for the lineage markers CD34 and CD45, and strongly positive for the specific surface antigens CD29 (95.3 ± 3.2%) and CD90 (94.9 ± 3.7%) (Figure 1B,C), which are typical of MSCs.

### Expression of exogenous angiopoietin-1 in mesenchymal stem cells

**Figure 2:**
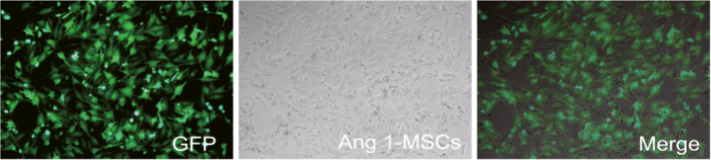
**Expression of green fluorescent protein in mesenchymal stem cells after angiopoietin-1 gene modification.** Ang1-MSCs, angiopoietin-1 gene-modified bone marrow mesenchymal stem cells; GFP, green fluorescent protein.

**Figure 3:**
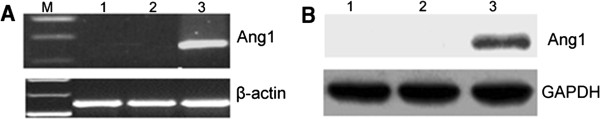
**Expression of exogenous angiopoietin-1 in mesenchymal stem cells. (A)** RT-PCR-amplified mRNA of exogenous gene. **(B)** Western blot analysis of exogenous proteins. Lane 1, unmodified mesenchymal stem cells (MSCs); lane 2, MSCs infected with control adenovirus; lane 3, MSCs infected with recombinant adenovirus containing Ang1-MSCs. GAPDH, glyceraldehyde 3-phosphate dehydrogenase.

We constructed recombinant adenovirus vectors expressing Ang1 and used them to infect MSCs. After infection, 96.4 ± 2.3% of the target cells expressed GFP (Figure 2), which is an indicator of efficient transfection. The RT-PCR products for the expected Ang1 fragment were detected in Ad-GFP-Ang1-MSCs (Figure 3A), but not in unmodified MSCs or Ad-GFP-MSCs (control adenovirus-infected MSCs). Consistent with the mRNA expression, Ang1 protein was only present in Ang1-MSCs (Figure 3B) and was not detected in MSCs or Ad-MSCs. These results indicate that transgenic Ang1 was successfully expressed in Ang1-MSCs.

### Angiopoietin-1 gene-modified bone marrow mesenchymal stem cells enhance wound healing

**Figure 4:**
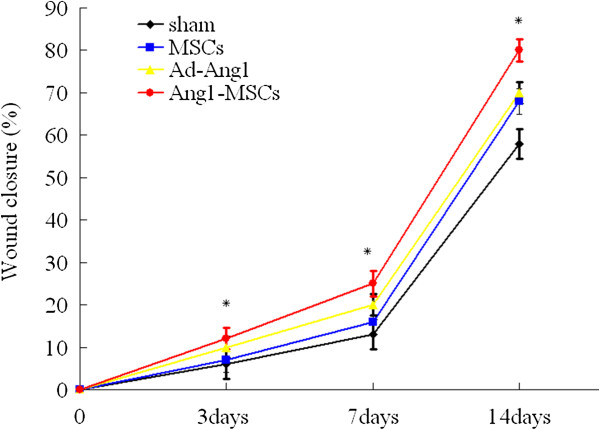
**Angiopoietin-1 gene-modified bone marrow mesenchymal stem cells promote wound closure *in vivo*.** Angiopoietin-1 gene-modified bone marrow mesenchymal stem cell (Ang1-MSC)-treated wounds healed faster compared with those in the other groups. Analysis of variance versus mesenchymal stem cells (MSCs), recombinant adenovirus encoding angiopoietin-1 (Ad-Ang1) or sham, *P <0.05.

Wound closure percentages in the different groups at different times post transplantation are shown in Figure 4. The wound closure percentage in the Ang1-MSC-treated rats was significantly greater than that in the other groups at different days post transplantation, suggesting that Ang1-MSCs accelerated wound closure compared with MSCs and Ad-Ang1.

### Histopathological evaluations

**Figure 5:**
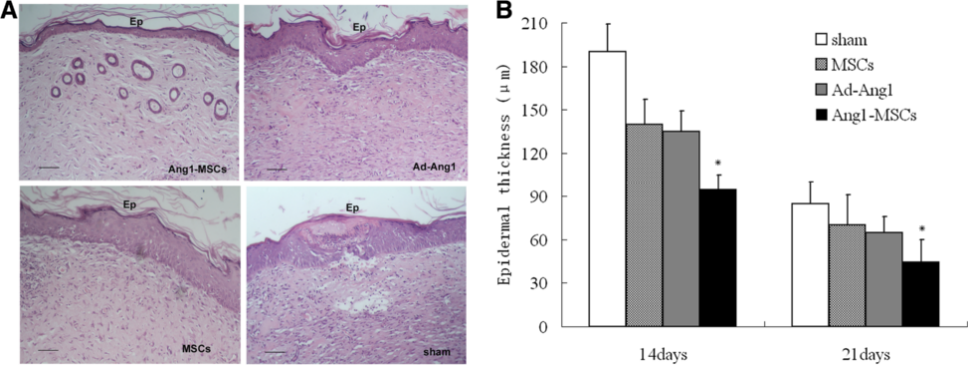
**Histological analysis of wound and epidermal thickness. (A)** Angiopoietin-1 gene-modified bone marrow mesenchymal stem cell (Ang1-MSC)-treated wounds showed more stable features in the granular and spinous layers, and the epidermal layer was more mature. Completely developed epidermis with regenerated skin appendages (arrows) is observed at day 21 after Ang1-MSC treatment of the wound. Scale bar = 50 μm. **(B)** Wounds treated with Ang1-MSCs displayed thinner epidermis compared with wounds treated in the other groups. Analysis of variance versus mesenchymal stem cells (MSCs), recombinant adenovirus encoding angiopoietin-1 (Ad-Ang1) or sham, *P <0.05.

Histological analysis over time indicated that Ang1-MSC-treated wounds showed more stable features in the granular and spinous layers and displayed a flatter epithelium with a more clearly developed stratum corneum (Figure 5A) compared with the other groups, indicating a more mature epithelium. In addition, Ang1-MSC-treated wounds appeared to have increased numbers of skin appendages compared with other groups. Moreover, the epidermal cellularity in the Ang1-MSC-treated wounds was significantly reduced and the epidermis was thinner (Figure 5B) compared with the other groups. At day 21, the epithelial thickness of the Ang1-MSC-treated wound equaled the thickness of the surrounding unwounded epithelium. However, epidermal cellularity in other-treated wounds was still higher and had a proliferative epithelium at day 21, suggesting higher cellular activity and prolongation of epidermal inflammation compared with Ang1-MSC-treated wounds. These results indicate that Ang1-MSCs accelerate epidermal regeneration.

### Collagen deposition

**Figure 6:**
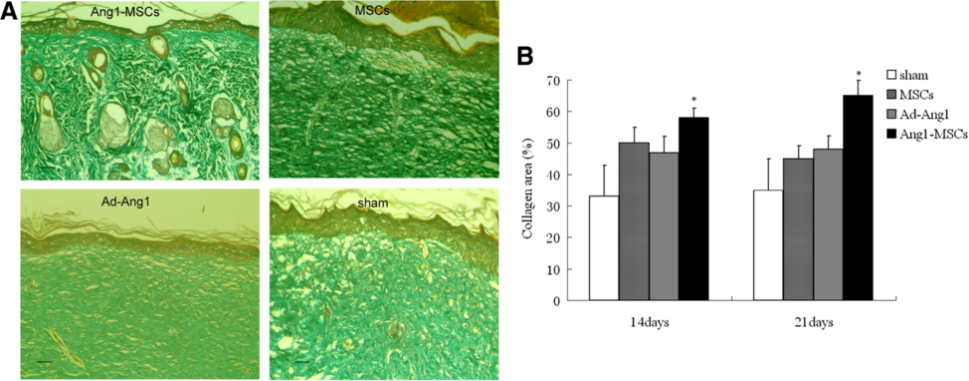
**Masson’s trichrome staining for collagen. (A)** Representative trichrome-stained images at day 21 after treatment. Angiopoietin-1 gene-modified bone marrow mesenchymal stem cell (Ang1-MSC)-treated wounds showed an arranged collagen network, indicating more mature collagen development than in the other groups. Scale bar = 50 μm. **(B)** Ang1-MSC-treated wounds display much denser collagen than wounds in the other groups. Analysis of variance versus mesenchymal stem cells (MSCs), recombinant adenovirus encoding angiopoietin-1 (Ad-Ang1) or sham, *P <0.05.

The results of Masson’s trichrome staining showed that Ang1-MSC-treated wounds displayed an arranged collagen network and smooth collagen deposition (Figure 6A), both of which were absent in the other groups, suggesting that Ang1-MSC-treated wounds had more mature collagen development than wounds in the other groups. We then measured the positive collagen-staining area relative to total area in the samples. The positive collagen staining percentage in the Ang1-MSC-treated wound was significantly greater than in the other groups at different days post transplantation (Figure 6B). The results showed that treatment with Ang1-MSCs significantly increased collagen deposition, resulting in much denser collagen than in any other treatment group, especially at day 21 after treatment.

### Engraftment of Ang1-MSCs into the wound skin

**Figure 7:**
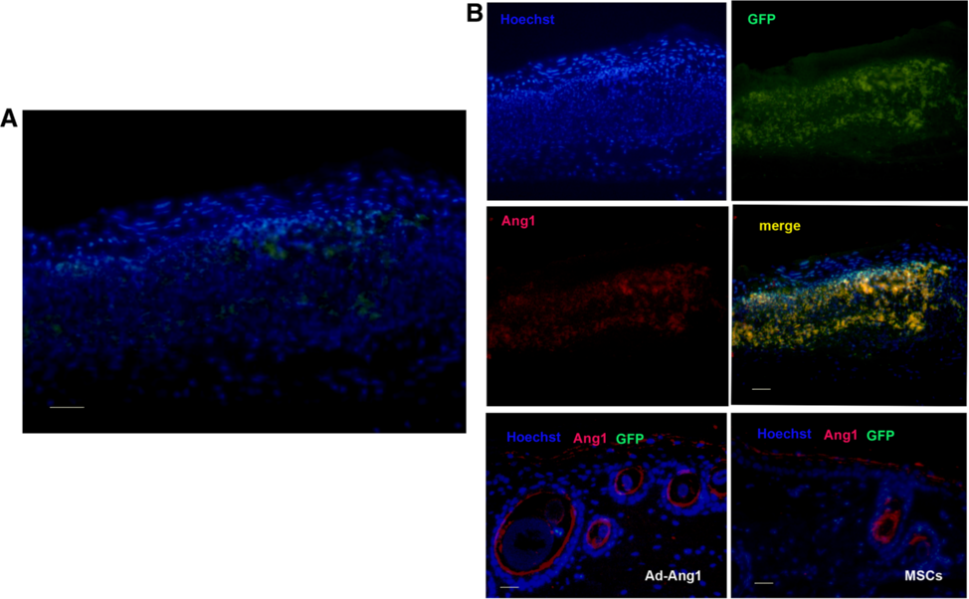
**Engraftments of angiopoietin-1 gene-modified bone marrow mesenchymal stem cells into wounded skin and angiopoietin-1 expression. (A)** Confocal microscopy images showing green fluorescent protein-positive (GFP^+^) mesenchymal stem cells (MSCs; green) in angiopoietin-1 gene-modified bone marrow mesenchymal stem cell (Ang1-MSC)-treated wounds at day 21 after transplantation. Nuclei were stained with Hoechst (blue). Scale bar = 50 μm. **(B)** Confocal microscopy images showing immunostaining for angiopoietin-1 (Ang1; red) in GFP-labeled MSCs (green) at day 7 after transplantation. Nuclei were stained with Hoechst (blue). After merging, the cells of coexpressing GFP and Ang1 are indicated (yellow). Scale bar = 50 μm. Ad-Ang1, recombinant adenovirus encoding angiopoietin-1.

Engraftment of Ang1-MSCs into the wound skin was detected by labeling gene-modified MSCs with GFP and observing them under a fluorescence microscope. Numerous GFP^+^ MSCs were found closely associated with host cells in Ang1-MSC-treated wounds at day 7, and GFP^+^ MSCs were seen in the epidermal and dermal components throughout the healing process even at day 21 after transplantation (Figure 7A). Cells of coexpressing GFP and Ang1 were detected in the wounds treated with Ang1-MSCs (Figure 7B) up to day 21 post treatment. These results indicate that topically applied Ang1-MSCs migrated into the wound, integrated into the neoepidermis and neodermis, and continually expressed Ang1.

### Angiopoietin-1 gene-modified bone marrow mesenchymal stem cells enhance angiogenesis

**Figure 8:**
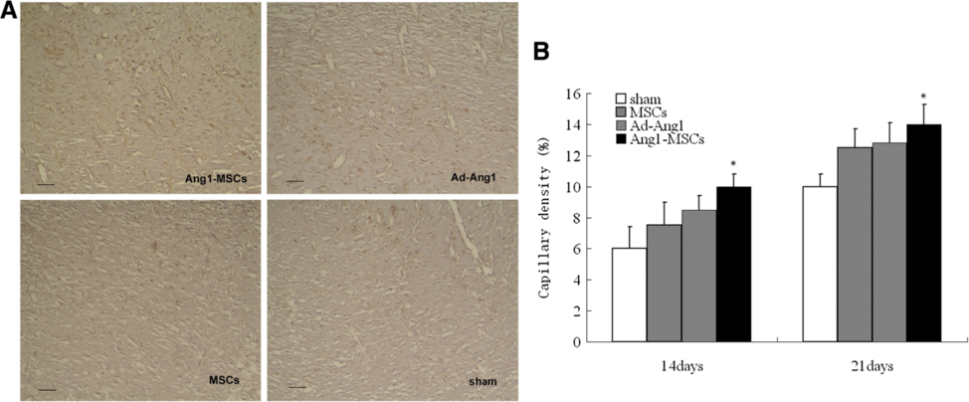
**Angiopoietin-1 gene-modified bone marrow mesenchymal stem cells promote angiogenesis. (A)** Representative images showing CD31 staining (yellow) on day 7 after wound treatment with angiopoietin-1 gene-modified bone marrow mesenchymal stem cells (Ang1-MSCs), recombinant adenovirus encoding angiopoietin-1 (Ad-Ang1), mesenchymal stem cells (MSCs), and sham. Scale bar = 50 μm. **(B)** Capillary density was increased in Ang1-MSC-treated wounds compared with MSC-treated, Ad-Ang1-treated, and sham-treated wounds. Analysis of variance versus MSCs, Ad-Ang1 or sham, *P <0.05.

**Figure 9:**
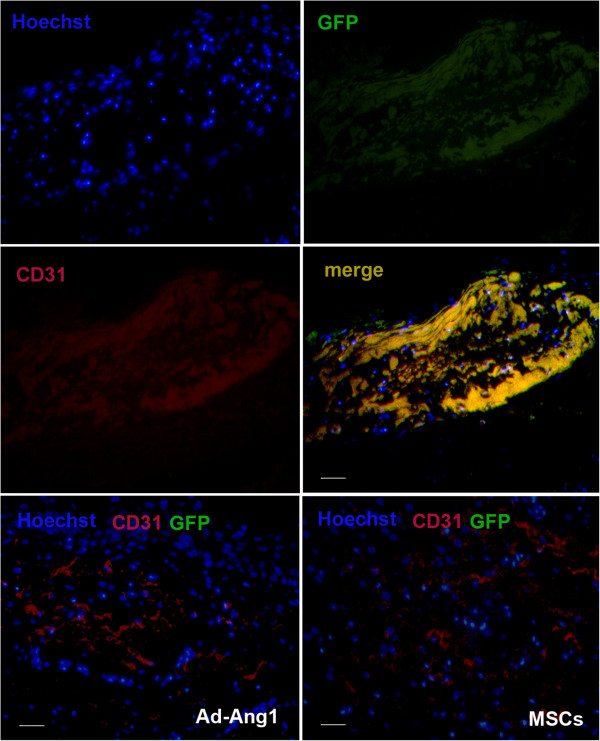
**Immunofluorescent staining for endothelial protein CD31.** Representative confocal images showing CD31 (red) and green fluorescent protein (GFP) staining at day 14 in a wound after treatment with recombinant adenovirus encoding angiopoietin-1 (Ad-Ang1), mesenchymal stem cells (MSCs) and angiopoietin-1 gene-modified bone marrow mesenchymal stem cells (Ang1-MSCs). Nuclei were stained blue with Hoechst (blue). GFP and CD31 double-positive cells (yellow) were only found in Ang1-MSC-treated wounds. Scale bar = 50 μm.

We performed immunostaining using a specific antibody for the blood vessel endothelial cell marker CD31. As shown in Figure 8A, the proportion of CD31-positive cells was significantly increased in Ang1-MSC-treated wounds compared with Ad-Ang1-treated, MSC-treated or sham-treated wounds. In addition, we measured overall blood vessel density (CD31-immunopositive areas/total areas) in the wound margin. The results showed the overall capillary density in the margin of Ang1-MSC-treated wounds was significantly higher than in that of the Ad-Ang1-treated, MSC-treated, or sham-treated wounds (Figure 8B). Moreover, to investigate the possibility that Ang1-MSCs enhance angiogenesis through differentiation into endothelial cells, we found cells that coexpressed CD31 and GFP (Figure 9), revealing that Ang1-MSCs were able to differentiate into endothelial cells. These observations suggest that the Ang1-MSC-induced improvements in wound healing occurred by enhanced angiogenesis, and that this enhanced angiogenesis resulted from differentiation of MSCs into endothelial cells.

## Discussion

Chronic skin wounds such as diabetic ulcers, radiation-induced injury-impaired wounds, and malnutrition-associated wounds are difficult to heal, and remain a great challenge to clinicians [27,28]. Little improvement has been made in the enhancing wound healing in the past few decades. Innovative treatments to enhance the chronic wound healing process are therefore needed.

Cell therapy has been applied to chronic wounds for many years. However, in previous studies, transplantation of some cells such as allogeneic fibroblasts just resulted in tissue repair, leading to increased inflammation and scar formation [29]. In this study, by tracing GFP expression, we observed transplanted Ang1-MSCs localized into the skin epithelium and dermis, and the GFP^+^ MSCs were still present up to day 21 after transplantation. Ang1-MSCs promoted epithelial maturation and collagen deposition, and significantly increased the number of regenerating appendage structures compared with MSC-treated, Ad-Ang1-treated, or sham-treated wounds. These results suggest that engrafted Ang1-MSCs in the wound accelerate epidermal and dermal regeneration rather than forming scar tissue.

Because of rapid degradation and drying out in the open wound, the topical application of various cell factors to stimulate chronic wound healing has been explored, but without significant advancements. Considerable attention has thus been paid to cell and gene therapies to treat chronic wounds. The combination of cell and gene therapies has been used to introduce various exogenous genes into chronic wounds in animal models, with the aim of overcoming the limitations of direct application of recombinant cell factors. In this study, Ang1 gene-modified MSCs were established by recombinant adenovirus infection and locally transplanted to the wound site. In our experiments, the efficiency of wound healing and skin regeneration was in the following order: Ang1-MSCs > MSCs or Ad-Ang1 > sham. Our results thus showed that the combination of MSCs with Ang1 gene therapy was more effective than the transplantation of either MSCs alone or Ad-Ang1 treatment alone. The combination of MSCs and Ang1 demonstrated synergistic effects on promoting wound repair, resulting from the effects of MSCs and the additional benefit of Ang1 expression. Ang1 produced by MSCs plays a role in coordinating and regulating cellular processes and wound healing.

Neovascularization is a crucial step in wound-healing process. In this study, we demonstrated that Ang1-MSC-treated wounds are surrounded by a denser capillary network compared with the other groups, suggesting that Ang1-MSCs promoted angiogenesis in the regenerated dermis compared with MSCs, Ad-Ang1 or sham. Our results also demonstrated that the engrafted GFP^+^ MSCs in the wound express CD31 subunit, and these GFP^+^CD31^+^ MSCs remained in the wound at day 21 after transplantation. These results suggest that Ang1-MSCs can thus differentiate into vascular endothelial cells in wounds, which may be partially responsible for the role of Ang1-MSCs in enhancing angiogenesis.

## Conclusion

This study demonstrated the efficacy of cell-based gene therapy with Ang1-MSCs on enhancing wound healing in rats by promoting epidermal and dermal regeneration, and angiogenesis. Ang1-MSCs administration may thus represent a novel therapeutic approach to clinically explore further improvements in cutaneous wound therapy.

## Abbreviations

Ad-Ang1: Recombinant adenovirus encoding Ang1; Ang1: Angiopoietin-1; Ang1-MSC: Angiopoietin-1 gene-modified bone marrow mesenchymal stem cell; DMEM: Dulbecco’s modified eagle’s medium; GFP: Green fluorescent protein; mAb: Monoclonal antibody; MSC: Mesenchymal stem cell; RT-PCR: Reverse transcription-polymerase chain reaction.

## Competing interests

The authors declare that they have no competing interests.

## Authors’ contributions

YLL carried out most of the study and drafted the manuscript. HYJ participated in the study design and helped to draft the manuscript. FZ, SHZ and YL carried out histological evaluation, the immunoassays and cell culture. XX, LLS, and YL participated in animals’ study and data discussion. WL participated in construction of skin wounds. SHZ, LZ, and XX participated in reconstruction of adenovirus vectors and gene transfection. LZ and FZ participated in collection of the data and performed the statistical analysis. All authors read and approved the final manuscript.
